# Case Report: Robot-assisted laparoscopic bladder diverticulectomy: a case series and initial experience

**DOI:** 10.3389/fsurg.2024.1453883

**Published:** 2025-01-06

**Authors:** Guangju Ge, Haihong Wang, Qiming Zheng, Shuai Zhang, Huan Wang, Liang Ma

**Affiliations:** ^1^ Department of Urology, Sir Run Run Shaw Hospital, Zhejiang University School of Medicine, Hangzhou, China; ^2^ Department of Anesthesiology, Sir Run Run Shaw Hospital, Zhejiang University School of Medicine, Hangzhou, China

**Keywords:** robot-assisted, bladder, diverticulectomy, tumor, experience

## Abstract

**Objectives:**

To explore the experience of tumor control technique in robot-assisted laparoscopic bladder diverticulectomy (RALBD) in the treatment of bladder diverticulum tumor, intraoperative tumor control and postoperative comprehensive treatment.

**Patients and methods:**

We treated three male patients with bladder diverticulum tumors. Case 1 involved a 63-year-old with a 3.0 cm tumor in the diverticulum on the right bladder wall. Case 2 involved a 70-year-old with a 1.0 cm cauliflower-like tumor in the diverticulum on the left bladder wall. Case 3 involved a 64-year-old with a 3.0 cm tumor in the diverticulum on the right bladder wall. Each patient underwent robot-assisted laparoscopic partial cystectomy (PC) with ureteral Double J (D-J) stent placement. To minimize the risk of intraoperative tumor spread, we implemented enhanced surgical techniques. Systemic chemotherapy and adjuvant intravesical chemotherapy were recommended to reduce the risk of tumor recurrence and metastasis.

**Results:**

Postoperative pathology confirmed papillary urothelial carcinoma in all three cases. Each patient was followed up for over 20 months, with no evidence of recurrence or distant metastasis observed through cystoscopy and chest and abdominal CT scans.

**Conclusion:**

For patients with urothelial carcinoma in a bladder diverticulum, robot-assisted laparoscopic bladder-sparing surgery is a viable option when appropriate cases are selected. Effective intraoperative tumor control and comprehensive postoperative treatment are crucial to minimizing recurrence and metastasis risks. The robotic approach offers enhanced precision and visualization compared to traditional open or laparoscopic techniques, potentially leading to improved outcomes regarding intraoperative tumor control and reduced postoperative complications. However, this study is limited by its small sample size of only three patients and short-term follow-up. A larger sample of patients is needed to confirm the advantages of the robotic approach.

## Introduction

Bladder cancer is the most common urinary tract malignancy, with at least 1% of cases occurring within a diverticulum (1, 2). A bladder diverticulum is a herniation of the bladder mucous membrane lacking a muscular layer, resulting in loss of muscle contractility and urinary stasis (3). Congenital diverticulum, caused by developmental defects in bladder muscle during embryogenesis, occur in approximately 1.7% of children and are not associated with lower urinary tract obstruction (4). The absence of a muscular layer in diverticula contributes to urinary stasis, which may lead to chronic inflammation, recurrent infections, and the development of dysplasia or metaplasia (5, 6). Historical series have indicated that the survival rates for urothelial carcinoma originating from bladder diverticula are lower compared to those of bladder tumors not arising from a diverticulum (7). However, more recent studies have shown that survival rates for patients with bladder diverticulum tumors are comparable to those of individuals with non-diverticular bladder cancer (8, 9). Despite this progress, data regarding the treatment and outcomes of bladder diverticulum tumors remain limited and primarily derive from small, single-center studies.

Surgical resection of bladder diverticula can be achieved through various approaches, including open surgery, endoscopy, laparoscopic (extraperitoneal or intraperitoneal), and robotic-assisted techniques (10–13). Traditional open surgery, often the primary treatment for bladder diverticulum tumors, or total cystectomy, can lead to significant patient morbidity and a reduced quality of life. Kees group concluded that there were no differences in overall survival (OS) or metastasis-free survival (MFS) between partial cystectomy and radical cystectomy groups for the bladder diverticulum tumors. In this study, five-year OS after radical cystectomy (RC) and partial cystectomy (PC) was 62% and 66%, respectively, while five-year MFS was 66% and 55% respectively. Therefore, PC may represent a feasible surgical alternative to RC in selected patients with bladder diverticulum tumors (14). Robotic technology enables more precise dissection and tumor removal while performing the PC, thanks to its high-definition 3D cameras and magnified visualization of the surgical area. With the widespread adoption of robotic technology, RALBD has advantages such as reduced blood loss, reduced postoperative morbidity, shorter hospital stays, quicker recovery times, and minimal scarring while maintaining effective oncological outcomes equivalent to those achieved via open surgery (15).

Bladder suturing and complete tumor excision are the main current surgical challenges for bladder diverticulum tumors. Previous reports on laparoscopic partial cystectomy have highlighted the utilization of stapling devices, such as the Endo-GIA (Covidien, Mansfield, MA), to facilitate the laparoscopic closure of the bladder. However, the use of such stapling instruments carries a significant theoretical risk of stone formation due to potential staple migration within the bladder. In contrast, robotic-assisted surgery enhances the surgeon's visibility and precision, allowing for the execution of bladder suturing in a controlled, two-layer technique, closely replicating the approach used in open surgery. More, with its magnified visual capabilities, RALBD allows for precise excision of the diverticulum, thereby minimizing the risk of residual tumor tissue. In this study, we explored feasibility, safety, and reproducibility of RALBD for treating bladder diverticulum tumors, gaining preliminary experience in intraoperative tumor control and comprehensive postoperative care. Furthermore, we report on perioperative, oncological, functional, and outcomes in patients undergoing RALBD.

## Patients and methods

The three cases were involved in this study. The inclusion criteria for selecting cases were as follows: (1) Diagnosed with bladder diverticulum tumors, confirmed by imaging studies such as ultrasound, CT, or MRI. (2) Patients were willing to undergo robotic surgery. (3) Patients with no other advanced or metastatic cancer and severe comorbidities that would preclude surgical intervention.

**Figure 1:**
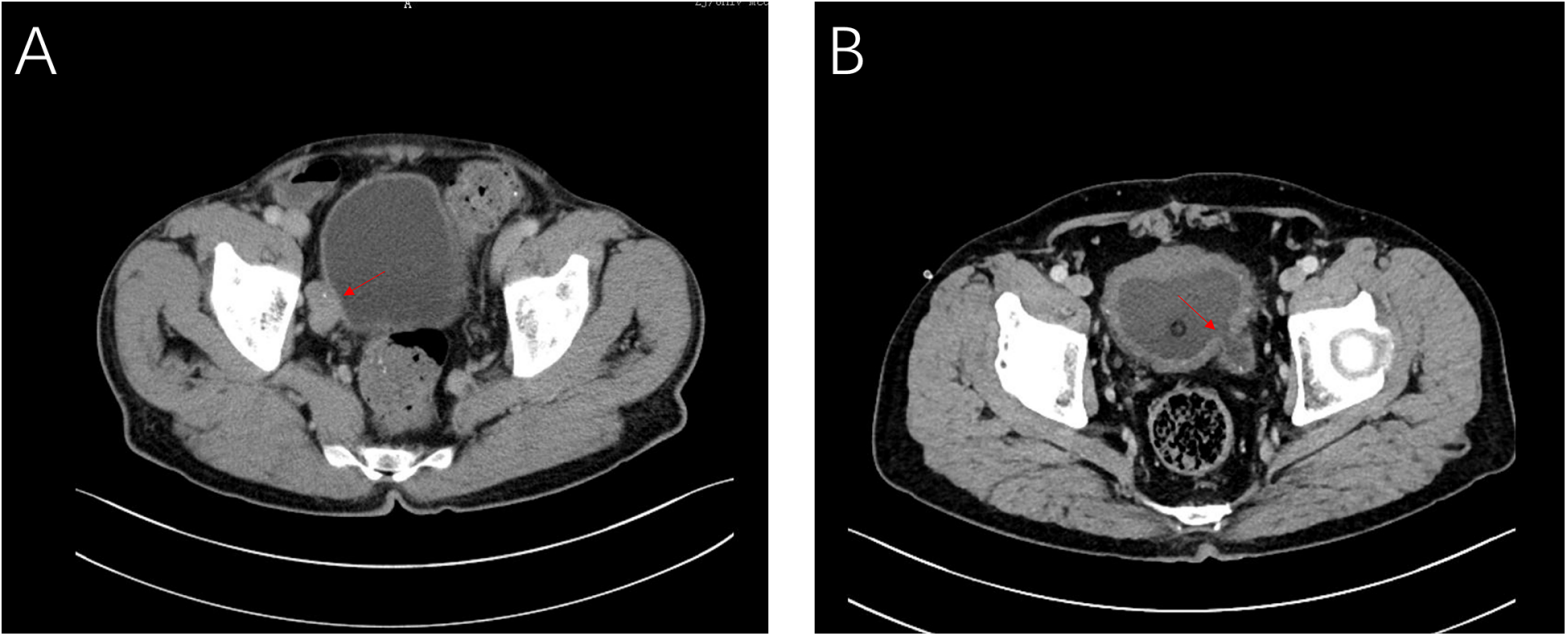
CT images of case 1 **(A)** and case 2 **(B)** showing the intradiverticular bladder tumor noted with arrow.

The first patient was a 63-year-old man, admitted with the complaint of “repeated gross hematuria for one week”. Further abdominal enhanced CT displayed space-occupying lesion on the right wall of bladder (Figure 1A), considering tumor. Cystoscopy displayed a large diverticulum filled with tumor on the right wall of the bladder. Biopsy was performed. Pathology examination displayed low level (bladder) papillary urothelial carcinoma.

The second patient was a 70-year-old man, admitted with the complaint of “dysuria for one month”. Cystoscopy and abdominal CT (Figure 1B) revealed that a bladder tumor about 2.0 cm in size could be seen in the diverticulum on the left wall of bladder. Biopsy and pathology revealed (bladder) papillary urothelial tumor with low malignant potential.

**Figure 2:**
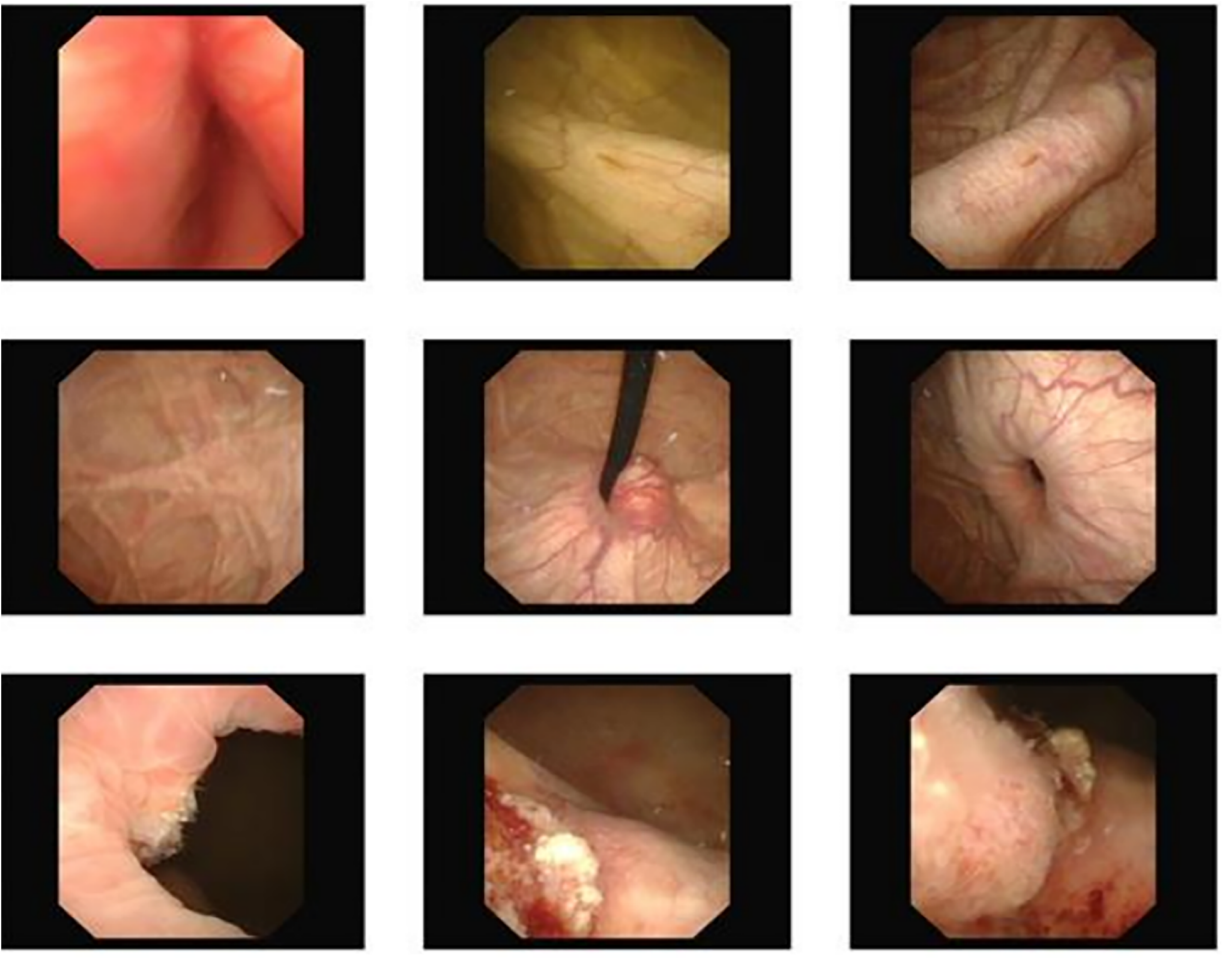
Cystoscopy showed a large diverticulum on the right wall of the bladder.

The third patient was a 64-year-old man, admitted with the complaint of “gross hematuria for ten days”. Further cystoscopy displayed space-occupying lesion on the right wall of bladder (Figure 2).

**Figure 3:**
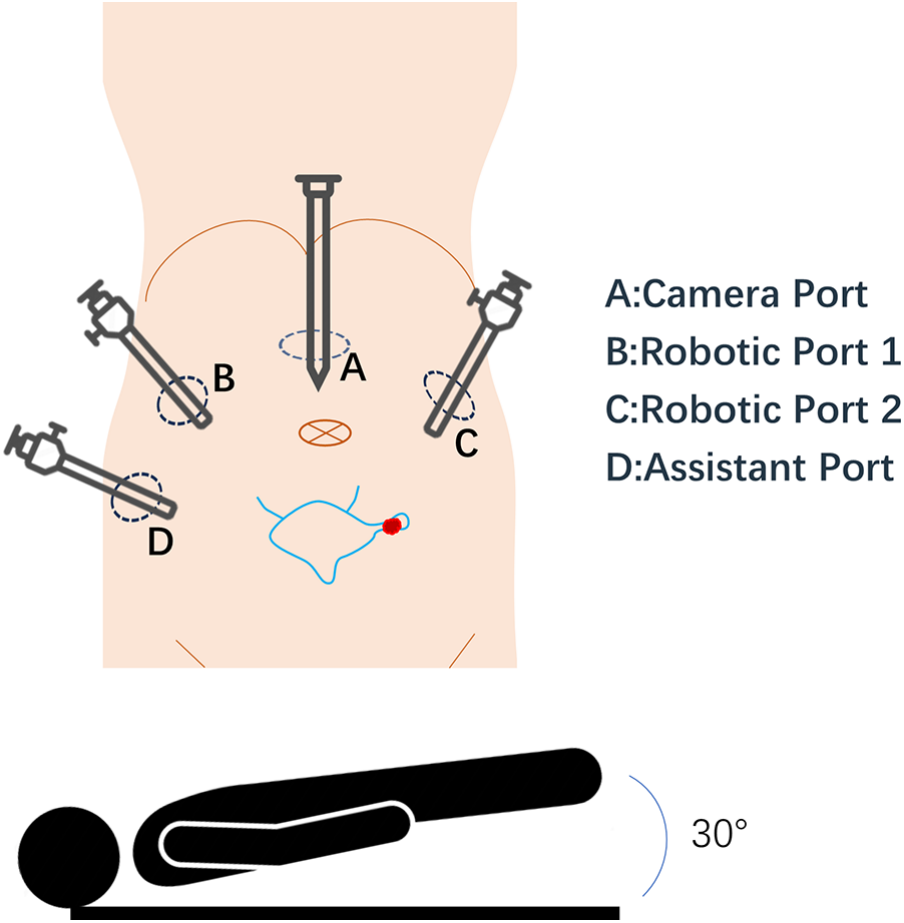
Robotic-assisted surgical setup for bladder diverticulum resection. This schematic diagram illustrates the positioning of the patient and placement of robotic ports during surgery. The diagram highlights the bladder and diverticulum. Port placements are indicated by circles labeled (**A**), (**B**), (**C**), (**D**).

**Figure 4:**
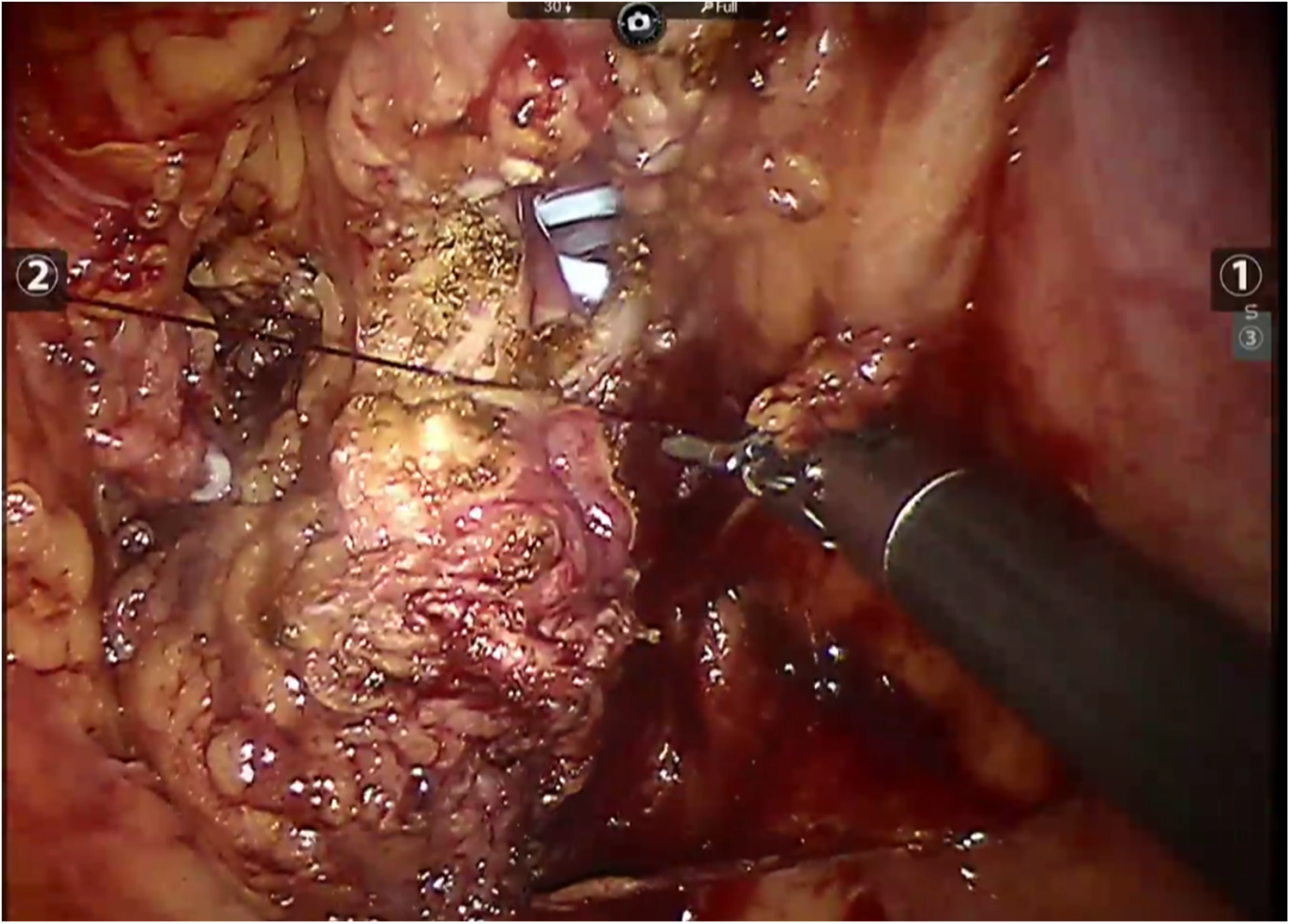
Robot-assisted closure of the bladder incision in case 2.

All patients underwent robot-assisted laparoscopic partial cystectomy and ureteroscopy D-J tube indwelling. Following the successful administration of anesthesia, patients were positioned in a low lithotomy position with a 30-degree Trendelenburg tilt. A 6 Fr ureteral stent was inserted into the ureter on the tumor side to protect the ureteral orifice during the procedure, and a 16 Fr catheter was placed for urinary drainage. The DaVinci robot system was put into position and installed after routine disinfection and towel laying. A skin incision was made approximately 2.0 cm above the umbilicus, and a pneumoperitoneum was established with a Veress needle at a pressure of 15 mmHg, followed by the placement of a 12 mm trocar. Under the surveillance of the camera, puncture was performed at the level of the lower edge of the umbilical of the mid-clavicular line on the left and right sides and at the McBurney point, and 12 mm, 12 mm and 8 mm Trocar were placed (Figure 3). The bladder was filled with distilled water through the catheter. This approach not only reduces the presence of free tumor cells in the bladder but also facilitates the identification of bladder diverticulum during the procedure. The bladder diverticulum was identified, and the bladder was emptied prior to making an incision in its wall. The catheter was maintained unobstructed to ensure continuous drainage, creating a relative negative pressure within the bladder. Upon opening the bladder, it was observed that the diverticulum in the first patient was small, with a diameter of approximately 1.5 cm. Before resection, the mucosa of the diverticulum was sutured and the diverticulum was closed to reduce the risk of tumor dissemination. In the second case, given the large size of the diverticulum, both the diverticulum and tumor inside were completely removed (Figure 4). The specimens after resection were directly packed into the specimen bag to avoid the contamination in the abdominal cavity. The bladder is sutured continuously with 3-0 absorbable threads. After closing the peritoneum incision, a catheter was inserted with 20 ml normal saline injected into the catheter balloon. Up to 150 ml normal saline was injected into the bladder with no visible leakage. The specimen was taken out from the pelvic cavity. Following thorough irrigation and aspiration of the wound, a pelvic drainage tube was inserted. The incision and puncture sites were then sutured, and the procedure was successfully completed. The patients were treated with systemic chemotherapy and intravesical chemotherapy after operation. All patients received 4 cycles of the GC chemotherapy regimen includes gemcitabine and cisplatin.

This schematic diagram illustrates the positioning of the patient and placement of robotic ports during surgery. The diagram highlights the bladder and diverticulum. Port placements are indicated by circles labeled A, B, C, D.

This study was approved by the Institutional Ethics Committee of Sir Run Run Shaw Hospital in Hangzhou, China. Written informed consent was obtained from the three patients.

## Results

There was no complication in all the cases. The drainage tube was removed one week after surgery, the catheter two weeks postoperatively, and the D-J stent after two months. Table 1 shows pathology operative metrics, and outcomes of all patients. Postoperative pathology of the first patient displayed low grade non-invasive papillary urothelial carcinoma with glandular differentiation and urothelial hyperplasia on both sides of the cutting edge but negative basal cutting edge. No recurrence was found 27 months after operation. Postoperative pathology of the second patient displayed high grade papillary urothelial carcinoma with local invasion of lamina propria. No tumor recurrence was found 21 months after operation. Postoperative pathology of the third patient displayed invasive urothelial carcinoma with focal glandular differentiation. No tumor recurrence was found 16 months after operation.

**Table 1:** Clinical features of patients undergoing RALBD.

Patient	Age (years)	Gender	Tumor size (cm)	Pathology	Duration of surgery time (min)	Estimated Blood Loss (ml)	Follow-up (months)	Complication
1	63	Male	1.2	Low grade non-invasive papillary urothelial carcinoma with glandular differentiation	90	50	27	None
2	70	Male	2.0	High grade papillary urothelial carcinoma with local invasion of lamina propria	110	50	21	Urinary retention
3	64	Male	1.0	Invasive urothelial carcinoma with focal glandular differentiation	200	100	16	None

The bladder diverticulum was noted to vary in size, with some being larger than expected, complicating the surgical approach. Larger diverticula posed challenges in ensuring complete resection and preventing damage to adjacent structures. The second patient developed minor postoperative complication. Due to benign prostatic hyperplasia, the patient had trouble urinating after the catheter was remove postoperatively. To prevent diverticulum recurrence, the patient subsequently underwent a transurethral resection of the prostate (TURP) procedure.

## Discussion

Bladder cancer is the most prevalent urinary tract malignancy, with bladder diverticula potentially increasing the risk of tumor development. Studies suggest that the presence of diverticula raises the likelihood of tumor formation by 0.8%–10% compared to a normal bladder (16). Tumors within diverticula can be detected using ultrasound, CT and magnetic resonance imaging (MRI). And CT features of diverticulum tumor can predict clinical outcomes (17). Recently, Panebianco et al. (18) proposed that bladder MRI can supplement CT imaging. Endoscopy can be more effective in finding lesions and obtaining biopsy to make a clear diagnosis.

Radical cystectomy (RC) is a well-established treatment for diverticulum tumors (8), but bladder-sparing methods such as transurethral resection (TUR) or partial cystectomy have also been shown to be feasible and oncological safe (9). However, there are limited guidelines for treating bladder diverticulum tumors, aside from those provided by the French Urological Association, which recommends partial cystectomy with pelvic lymphadenectomy for single-focus tumors confined to a diverticulum (19). In carefully selected patients with diverticulum tumors, partial cystectomy may be a feasible alternative to radical cystectomy (14).

Since 2006, several reports have described RALBD for bladder diverticulum tumors in adult patients (Table 2). The operations were successful, and no recurrence was found in the half year follow-up. Three articles reported the treatment of malignant vertical tumor with RALBD. Tareen et al. (15) reported a case of high-grade transitional cell carcinoma (TCC) invading lamina propria in 2008. The patient was later found to have cancer *in situ* and began taking bacilli Calmette-Guérin (BCG) maintenance treatment. Alturnende et al. (20) reported another two cases in 2011. One case of high-grade urothelial carcinoma in the diverticulum and two lesions of carcinoma *in situ* were found in other parts of the bladder. The patient received bilateral pelvic lymph node dissection and subsequent intravesical immunotherapy. Their other patient had a low-grade non-invasive transitional cell carcinoma of the bladder, so he had a RALBD and simultaneous bipolar TURP. Sophie Elands et al. (21) described an 84-year-old man with TCC treated with RALBD and ureteral re-implantation in 2015, and no further treatment was adopted for this patient. If the diverticulum is close to the ureteral orifice, a ureteral stent or simultaneous ureteral disconnection and replantation may be required. Our mean operative time of 133 min is similar to that of than these researches, which ranges from 160 to 250 min. Furthermore, no recurrence was also observed in our study; however, the follow-up duration was longer compared with previous study. Another distinction lies in our use of systemic chemotherapy in place of lymph node dissection, which appears to have yielded comparable outcomes.

**Table 2:** Published cases of RALBD in adult patients since 2006.

First author	Year	*N*	Indication	Surgery	Dimension (cm)
Tareen	2008	2	lesion post TURBT for high-grade TCC	RALBD	/
			High grade TCC	RALBD	/
Altunrende	2011	2	high grade TCC	RALBD + B/L PLND	2.1 × 0.97
			low grade non-invasive TCC	RALBD + bipolar TURP	4.7 × 2.5
Sophie Elands	2015	1	high grade TCC	RALBD + ureteral re-implantation	6.5

RALBD, robot-assisted laparoscopic bladder diverticulectomy; PLND, pelvic lymph node dissection; TURP, transurethral resection of the prostate; TCC, transitional cell carcinoma.

Robotic laparoscopic surgery for bladder diverticulum tumor may lead to intraperitoneal tumor dissemination, mainly through urine, gas and surgical instruments. Previous studies have reported that the risk of intraperitoneal contamination of urine in bladder was increased by cystoscopy monitoring during robotic surgery. However, the Mohammed group, in a study of 28 patients with interdiverticular bladder tumors, recommended robot-assisted diverticulectomy for its ability to reduce morbidity and postoperative complications (22).

Robotic bladder diverticulectomy shows significant advantages in surgical precision and operational flexibility compared to open and traditional laparoscopic procedures (23–25). While open surgery provides more direct visualization, it is associated with higher trauma, longer recovery periods, and a greater risk of postoperative complications. In contrast, laparoscopic surgery reduces trauma but still faces limitations in operational flexibility and visualization. Robotic-assisted surgery, through its precise three-dimensional visualization and enhanced instrument articulation, improves surgical precision and allows for faster postoperative recovery.

In our experience with robot surgery, we also found that the magnified three-dimensional view and enhanced surgeon comfort offered by robotic surgery also apply effectively to bladder-preserving procedures. One of the notable challenges associated with robotic-assisted surgery is its high cost, both in terms of initial investment and ongoing maintenance. The robotic systems themselves are expensive, and the surgical consumables required for robotic procedures are typically more costly than those used in traditional surgeries. Additionally, the adoption of robotic surgery requires specialized training for surgeons and the surgical team. This process is both time-consuming and costly but essential for ensuring effective use of the technology. However, we believe that with the advancement of technology, the costs will decrease, and robotic surgery will be widely adopted, ultimately benefiting patients.

Three patients in our center were treated with robotic laparoscopic partial cystectomy for bladder diverticulum tumor. We adopted a series of improved surgical techniques to reduce intraoperative tumor dissemination:

1. Before opening the bladder, the bladder should be filled with distilled water to inactivate the exfoliated and scattered tumor cells.
2. Empty the bladder before opening it, and then open the bladder 2.0 cm away from the diverticulum to avoid direct contact with the tumor.
3. After opening the bladder, the catheter is in an open and deflated state to create a relative negative pressure in the bladder to avoid the air flow in the bladder through the abdominal cavity causing tumor cells to spread.
4. Before resection of diverticulum, the neck of diverticulum should be sutured to close the diverticulum to avoid tumor cell dissemination.
5. Take out tumor specimen with a bag to avoid contact between tumor and wound or abdominal cavity.
6. Clear operation field and accurate suture in robotic surgery makes it conducive to tumor control.
7. After resection of bladder diverticulum and tumor, the surgical instruments were taken out in time and cleaned before use to reduce tumor spread.

We didn't use any staple device because it is difficult to ensure that tumors were completely resected.

Additionally, the histologic heterogeneity of bladder cancer is associated with prognosis. While urothelial carcinoma is the most common histology, more than 25% of patients with bladder cancer harbor variant histology. These variants, including clear-cell, plasmacytoid, small-cell, and sarcomatous, are associated with a higher risk of upstaging and worse outcomes compared to pure urothelial carcinoma (26). In our study, we observed two patients with glandular differentiation in the diverticula. According to the Francesco group, glandular differentiation is not associated with disease-specific survival (27), and no recurrence has been observed in our two patients to date.

Although pelvic lymphadenectomy (PLND) is an integral part of RC, it has not been well established in bladder preserving therapy (14). PLND was the standard procedure in some centers, while in others, it was only performed when lymph nodes were suspected. As for the three cases in our group, preoperative imaging evaluation did not show any lymph node metastasis, so pelvic lymph node dissection was not performed.

TUR is technically challenging. The narrow neck of diverticulum might limit the access to tumors. In addition, due to the lack of muscle layer in the diverticulum wall, it is difficult for surgeons to judge the depth of the resection. We found that it would be difficult to perform TUR so partial cystectomy was carried in all cases.

Charlotte et al. reported the recurrence rates of RC group and PC group were 27/81 (33%) and 16/34 (47%) respectively, median time to recurrence was 8.0 months in the RC group and 12.1 months in the PC group (14). Although the follow-up period for our three patients has been less than five years, no recurrences have been observed so far.

In our study, the small sample size and the absence of a control group are two major limitations. Due to the limited number of participants, the external generalizability of the results may be affected. The lack of a control group prevents direct comparison of robotic surgery with other surgical methods. Therefore, future studies should expand the sample size and include appropriate control groups to better assess the advantages and shortcomings of robotic surgery.

## Conclusions

For urothelial carcinoma in bladder diverticulum, it's important to carefully select appropriate cases, ensure effective intraoperative tumor control, and provide thorough postoperative adjuvant treatment. Additionally, performing robot-assisted laparoscopic bladder-sparing surgery can be a viable option.

## Data Availability

The raw data supporting the conclusions of this article will be made available by the authors, without undue reservation.

## References

[1] Walker NF Gan C Olsburgh J Khan MS. Diagnosis and management of intradiverticular bladder tumors. Nat Rev Urol. (2014) 11:383–90. 10.1038/nrurol.2014.131 24934450

[2] Halaseh SA Halaseh S Alali Y Ashour ME Alharayzah MJ. A review of the etiology and epidemiology of bladder cancer: all you need to know. Cureus. (2022) 14(7):e27330. 10.7759/cureus.27330 36042998 PMC9411696

[3] Garat JM Angerri O Caffaratti J Moscatiello P Villavicencio H. Primary congenital bladder diverticula in children. Urology. (2007) 70(5):984–8. 10.1016/j.urology.2007.06.1108 18068458

[4] Blane CE Zerin JM Bloom DA. Bladder diverticula in children. Radiology. (1994) 190:695–7. 10.1148/radiology.190.3.8115613 8115613

[5] Abou Zahr R Chalhoub K Ollaik F Nohra J. Congenital bladder diverticulum in adults: a case report and review of the literature. Case Rep Urol. (2018) 2018:9748926. 10.1155/2018/9748926 29568661 PMC5820673

[6] Shah B Rodriguez R Krasnokutsky S Shah SM Ali Khan S. Tumour in a giant bladder diverticulum: a case report and review of literature. Int Urol Nephrol. (1997) 29(2):173–9. 10.1007/BF02551338 9241544

[7] Mićić S Ilić V. Incidence of neoplasm in vesical diverticula. J Urol. (1983) 129(4):734–5. 10.1016/S0022-5347(17)52332-0 6405057

[8] Hu B Satkunasivam R Schuckman A Miranda G Cai J Daneshmand S. Urothelial carcinoma in bladder diverticula: outcomes after radical cystectomy. World J Urol. (2015) 33:1397–402. 10.1007/s00345-014-1472-5 25549760

[9] Golijanin D Yossepowitch O Beck SD Sogani P Dalbagni G. Carcinoma in a bladder diverticulum: presentation and treatment outcome. J Urol. (2003) 170:1761–4. 10.1097/01.ju.0000091800.15071.52 14532771

[10] Orandi A. Transurethral fulguration of bladder diverticulum: new procedure. Urology. (1977) 10:30–2. 10.1016/0090-4295(77)90033-4 406705

[11] Parra RO Jones JP Andrus CH Hagood PG. Laparoscopic diverticulectomy: preliminary report of a new approach for the treatment of bladder diverticulum. J Urol. (1992) 148:869–71. 10.1016/S0022-5347(17)36748-4 1387421

[12] Nadler RB Pearle MS McDougall EM Clayman RV. Laparoscopic extraperitoneal bladder diverticulectomy: initial experience. Urology. (1995) 45:524–7. 10.1016/S0090-4295(99)80029-6 7879345

[13] Allaparthi S Ramanathan R Balaji KC. Robotic partial cystectomy for bladder cancer: a single-institutional pilot study. J Endourol. (2010) 24:223–7. 10.1089/end.2009.0367 20039797

[14] Voskuilen CS Seiler R Rink M Poyet C Noon AP Roghmann F Urothelial carcinoma in bladder diverticula: a multicenter analysis of characteristics and clinical outcomes. Eur Urol Focus. (2020) 6(6):1226–32. 10.1016/j.euf.2018.12.002 30559065

[15] Tareen BU Mufarrij PW Godoy G Stifelman MD. Robot-assisted laparoscopic partial cystectomy and diverticulectomy: initial experience of four cases. J Endourol. (2008) 22:1497–500. 10.1089/end.2007.0297 18690815

[16] Eksioglu AS. Transitional cell carcinoma within a bladder diverticulum: CT findings and pathologic correlation. Gazi Medical Journal. (2009) 20(3):131–34.

[17] Di Paolo PL Vargas HA Karlo CA Lakhman Y Zheng J Moskowitz CS Intra-diverticular bladder cancer: CT imaging features and their association with clinical outcomes. Clin Imaging. (2015) 39(1):94–8. 10.1016/j.clinimag.2014.10.004 25457532 PMC4268062

[18] Panebianco V Narumi Y Altun E Bochner BH Efstathiou JA Hafeez S Multiparametric magnetic resonance imaging for bladder cancer: development of VI-RADS (vesical imaging-reporting and data system). Eur Urol. (2018) 74(3):294–306. 10.1016/j.eururo.2018.04.029 29755006 PMC6690492

[19] Roupret M Neuzillet Y Masson-Lecomte A Colin P Compérat EM Dubosq F Recommandations en onco-urologie 2016–2018 du CCAFU: tumeurs de la vessie. Prog Urol. (2016) 27:S67–91. 10.1016/S1166-7087(16)30704-7 27846935

[20] Altunrende F Autorino R Patel NS White MA Khanna R Laydner H Robotic bladder diverticulectomy: technique and surgical outcomes. Int J Urol. (2011) 18:265–71. 10.1111/j.1442-2042.2010.02716.x 21299640

[21] Elands S Vasdev N Tay A Adshead JM. Robot-assisted laparoscopic bladder diverticulectomy and ureteral Re-implantation for a diverticulum containing high grade transitional cell carcinoma. Curr Urol. (2015) 8(2):104–8. 10.1159/000365699 26889127 PMC4748793

[22] Amer ML Mumtaz H Russell B Gan J Rehman Z Nair R Intra-diverticular bladder tumours: how to manage rationally. Soc Int D’Urol J. (2022) 3(5):303–13. 10.48083/JCLW6772

[23] Aijaz P Farooqi Baloch K Faiz H Durvesh AK Tirmizi SJ Khan M Clinical presentation, tumor characteristics, and management of intradiverticular transitional cell carcinoma of the urinary bladder: a systematic review. Cureus. (2024) 16(6):e62974. 10.7759/cureus.62974 38912078 PMC11194034

[24] Janardanan S Nigam A Moschonas D Perry M Patil K. Urinary bladder diverticulum: a single-center experience in the management of refractory lower urinary symptoms using a robotic platform. Cureus. (2023) 15(7):e42354. 10.7759/cureus.42354 37621793 PMC10445242

[25] Poletajew S Krajewski W Adamowicz J Kołodziej A Zdrojowy R Radziszewski P. Management of intradiverticular bladder tumours: a systematic review. Urol Int. (2020) 104(1-2):42–7. 10.1159/000503868 31851992

[26] McFadden J Tachibana I Adra N Collins K Cary C Koch M Impact of variant histology on upstaging and survival in patients with nonmuscle invasive bladder cancer undergoing radical cystectomy. Urol Oncol. (2024) 42(3):69.e11–e16. 10.1016/j.urolonc.2023.12.008 38267301

[27] Claps F van de Kamp MW Mayr R Bostrom PJ Shariat SF Hippe K Prognostic impact of variant histologies in urothelial bladder cancer treated with radical cystectomy. BJU Int. (2023) 132:170–80. 10.1111/bju.15984 36748180

